# Establishment of a cell culture model based on primary epithelial cells to investigate damage and repair of respiratory epithelial cells

**DOI:** 10.1186/1939-4551-8-S1-A215

**Published:** 2015-04-08

**Authors:** Eva E Waltl, Regina Maria Selb, Julia Eckl-Dorna, Verena Niederberger, Rudolf Valenta

**Affiliations:** 1 Medical University of Vienna, Austria

## Background

The nasal epithelium represents the first barrier against entry of airborne particles into the respiratory system and therefore protects against allergens, pollutants and pathogens. We have previously used the respiratory epithelial cell line 16HBE14o- to define factors which can impair the barrier function of the respiratory mucosa. However, cultures of primary epithelial cells obtained from human nasal biopsies should resemble the natural situation in the nose better. Here we investigated primary human nasal epithelial cells regarding growth characteristics and sensitivity to damage by interferon-gamma and other factors.

## Methods

Primary epithelial cells were isolated from nasal biopsies from allergic and non-allergic patients undergoing routine nasal surgery at the ENT Department of the General Hospital of Vienna with consent from the Ethics Committee of the Medical University of Vienna. Primary cells and 16HBE14o- cells were grown on semipermeable membranes, which allow exposure of the cells to various factors both from the apical and the basolateral side. Cells were grown until they reached confluence and gained a trans-epithelial resistance of more than 1000 Ohm. Cell layers were then exposed to interferon-gamma which is known to impair epithelial integrity. Furthermore, a scratch-test model was used to investigate epithelial repair after physical damage.

## Results

Expression of epithelial cell makers on the surface of cultivated primary cells was confirmed by flow cytometry analysis. Histological staining of cultivated cells isolated by cytospins showed normal morphology with apical cilia. Normal function of cultured primary epithelial cells was shown by video analysis and ciliary beat frequency measurements. Barrier function decreased after exposure of cells to low doses (1ng/ml) of interferon gamma both in the case of primary and 16HBE14o- cells. Decrease of the barrier function was time- and dose-dependent. Repair of primary epithelial cell layers could be shown in the scratch-test model.

## Conclusions

We established a cell culture model using both primary nasal epithelial cells from allergic and non-allergic individuals, in which we can investigate exogenous and subject-specific factors which affect epithelial integrity. Supported by projects of the Austrian Science Fund (FWF), the DK W 1248-B13 program MCCA and P4613, P4605.

